# Hydroxypropyl Methylcellulose—A Key Excipient in Pharmaceutical Drug Delivery Systems

**DOI:** 10.3390/pharmaceutics17060784

**Published:** 2025-06-16

**Authors:** Robert-Alexandru Vlad, Andrada Pintea, Cezara Pintea, Emőke-Margit Rédai, Paula Antonoaea, Magdalena Bîrsan, Adriana Ciurba

**Affiliations:** 1 Department of Pharmaceutical Technology and Cosmetology, Faculty of Pharmacy, George Emil Palade University of Medicine, Pharmacy, Science, and Technology of Targu Mures, 540142 Targu Mures, Romania; robert.vlad@umfst.ro (R.-A.V.); emoke.redai@umfst.ro (E.-M.R.); paula.antonoaea@umfst.ro (P.A.); adriana.ciurba@umfst.ro (A.C.); 2 Medicine and Pharmacy Doctoral School, George Emil Palade University of Medicine, Pharmacy, Science, and Technology of Targu Mures, 540142 Targu Mures, Romania; 3 Department of Drug Industry and Pharmaceutical Biotechnology, “Grigore T. Popa” University of Medicine and Pharmacy, 700115 Iasi, Romania; magdalena.birsan@umfiasi.ro

**Keywords:** hydroxypropyl methylcellulose, polymerization degree, viscosity, film-forming agent, binder

## Abstract

Hydroxypropyl methylcellulose (Hypromellose, HPMC) is a well-known excipient used in the pharmaceutical and nutraceutical fields due to its versatile physicochemical properties. HPMC (derived from cellulose and obtained through etherification) varies in polymerization degree and viscosity, factors that both influence its functional applications. Usually, an increased polymerization degree implies a higher viscosity, depending also on the amount of polymer used. Hypromellose plays a crucial role in solid dosage forms, serving as a binder in the case of controlled-release tablets, a film-forming agent in the case of orodispersible films and mucoadhesive films, and a release modifier due to its presence in different polymerization degrees in the case of extended or modified release tablets. However, its compatibility with other excipients and the active ingredient must be carefully evaluated to prevent formulation challenges via several analytical methods such as differential scanned calorimetry (DSC), Fourier Transformed Infrared spectroscopy (FT-IR), X-Ray Particle Diffraction (XRPD), and Scanning Electron Microscopy (SEM). This review explores the physicochemical characteristics, and diverse applications of HPMC, emphasizing its significance in modern drug delivery systems.

## 1. Introduction

Cellulose is a biodegradable polymer of natural origin composed of repeated units of glucose. It possesses adequate mechanical properties, biocompatibility, and is easily accessible, aspects that led to the polymer’s recognition in industries such as pharmaceuticals, food, textiles, the paper industry, and many more in recent years. Among its limitations, cellulose presents poor solubility in water and organic solvents, due to its hydrophilic nature and crystalline structure. By modifying the initial structure, well-known cellulose derivatives were created through the etherification of the hydroxyl groups: Methyl cellulose (MC), Ethyl cellulose (EC), Hydroxyethyl cellulose (HEC), Hydroxypropyl cellulose (HPC), hydroxypropylmethyl cellulose (HPMC), carboxymethyl cellulose (CMC), and sodium carboxymethyl cellulose (NaCMC). This improved the solubility profile, offering additional applications in the biomedical field. Among these, HPMC stands out as a tablet binder, film-coating, gelling, and encapsulating agent, making it one of the most versatile cellulose derivatives used in the pharmaceutical domain [1,2].

Hydroxypropyl methylcellulose (HPMC), a semi-synthetic cellulose ether, is one of the most commonly used cellulose derivatives. Its excellent film-forming properties, high stability, remarkable biocompatibility, and biodegradability make it commonly employed in the food industry, the pharmaceutical industry, and other industries [3]. Also known as hypromellose, HPMC has now been used in hydrophilic matrices for over 60 years [4].

Hydroxypropyl methylcellulose is derived from natural cellulose through etherification, where hydroxyl groups in the cellulose structure are replaced with hydroxypropyl and methyl groups. This alteration can improve cellulose solubility, viscosity, film-forming properties, and soothing effects [5].

HPMC has extensive applications in various pharmaceutical technologies, playing a crucial role in developing mucoadhesive formulations, ophthalmic preparations, and different types of controlled-release dosage forms. Due to its properties, large biocompatibility, and edible character, it can be used as a tablet binder, film-coating material, biological adhesive, gelling agent, as well as an encapsulation, suspending, and thickening agent [6].

The properties of HPMC are dependent on the degree of substitution and molecular weight [7]. The food industry utilizes HPMC as a thickener, emulsifier, and stabilizer in processed foods [8]. Additionally, it plays a crucial role in construction materials, such as tile adhesives and cement-based coatings, due to its ability to improve water retention and workability [9].

Previous studies have shown that HPMC is suitable for water-soluble drugs, loading large amounts of drugs, and is compatible with numerous active pharmaceutical ingredients (APIs) such as bupropion hydrochloride, diclofenac sodium, and acetaminophen [10]. HPMC’s biodegradability and non-toxic nature make it a sustainable and safe polymer, further expanding its applications in eco-friendly and biomedical materials as mentioned by multiple international regulatory bodies (United States Food and Drug Administration—US-FDA and European Chemicals Agency). Ongoing research continues to enhance its properties and develop new applications, solidifying its importance in various industrial and scientific fields. Collectively, these assessments reinforce the status of HPMC as a safe, multifunctional polymer across pharmaceutical, food, cosmetic, and industrial applications.

Considering that this review is concerned with different pharmaceutical, analytical, and medical aspects, the first step consisted of establishing the names of the chapters and the subchapters. As a result, the main keywords used were as follows: HPMC, Hypromellose, hydroxypropyl methylcellulose as film-forming agent, hydroxypropyl methylcellulose as coating agent, HPMC utility in the medical field, HPMC interactions with other excipients, and HPMC conditions for degradation. The databases used for collecting information for this review were PubMed, Google Scholar, and Web of Science.

## 2. Chemical Structure and Physicochemical Properties

**Figure 1:**
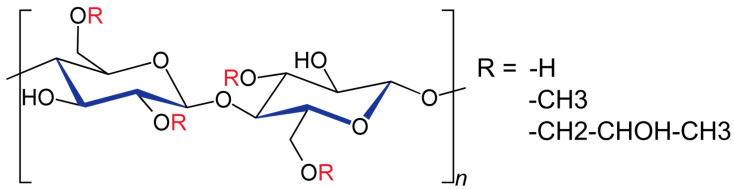
Chemical structure of hydroxypropyl methylcellulose.

Hypromellose (Hypromellosum—Ph. Eur. 11) (Figure 1) is a white, free-flowing, hygroscopic powder, and due to its lack of odour, and taste, as well as its edible character, it has been approved as a food additive by both the U.S. Food and Drug Administration agency and the European Union. The daily dosage accepted by the FDA for oral intake is 25 mg/kg [11,12]. In addition to its water-solubility, the non-ionic polymer is soluble in polar organic solvents and insoluble in pure chloroform or ethanol [13,14]. It is stable at pH values between 3 and 11 [15].

As previously mentioned, hydroxypropyl methylcellulose belongs to a group of semi-synthetic cellulose derivatives, whose hydroxyl groups have been partly substituted by ether-linked methoxy and hydroxypropyl side groups [16]. This addition disrupts cellulose’s crystalline structure, contributing to HPMC’s hydrophilic nature. Its unique character relies on the ability to hydrate, swell, and form a gel upon heating at 75–90 °C while being soluble in cold water [17,18].

For its synthesis, cellulose is first treated with an alkaline base such as NaOH. The etherification can be obtained through the chemical reaction with methyl chloride and propylene oxide, the latter resulting in chain extension [19,20,21]. The substitution of the glycosyl units occurs in positions 2, 3, or 6 with methyl or 2-hydroxypropyl as radicals [22]. Lismeri et al. attempted to produce HPMC from α-cellulose derived from cassava stems by using dimethyl sulphate for the methylation reaction [23]. Another method proposed by Yuan et al. suggests first methylating cellulose to obtain Methylcellulose (MC) and then synthesizing HPMC through hydroxypropylation [24]. Therefore, the polysaccharide-based polymer is available in a wide variety of substitution degrees, each differing in molecular weight, and presenting various features that directly impact the field of application [25]. For HPMC, the degree of substitution (DS) provides information on the number of methoxy groups substituted per repeated molecule, whereas molar substitution (MS) refers to the average hydroxypropyl molar content, the second-mentioned being the ability to undergo chain reactions, increasing the MS value. Depending on the character provided by the methoxy (hydrophobic) and the hydroxypropyl (hydrophilic) groups, the behaviour in aqueous media presents different particularities [26]. The ratio between the two substituents directly influences the compound’s solubility, swelling capacity, and thermal gelation temperature, dividing the field into subcategories [27]. A higher percentage of the methoxy substituent leads to an increase in hydrophobic interactions and, ultimately, a lower hydration rate, swelling, and matrix gel strength, through the obstruction of the hydrophilic groups [10]. On the other hand, a study carried out by Perez-Robles et al. investigated the impact of DS and MS on the connection between temperature and the gelation process. It was shown that with the increase of the DS and MS values, gelation took place faster, and the viscosity of the produced gel was lower [28].

The European Pharmacopoeia 11th edition [29] classifies hypromellose into four categories based on the substitution content, as presented in Table 1, where the first two digits represent the average percentage of the methoxy groups (1828), while the last two stand for the hydroxypropyl substituent percentage (1828). As part of the Methocel^®^ product line, Dow Chemical Company introduced the E, F, J, and K classification system, which dates back to the mid-20th century, but since 2019, it has belonged to DuPont [21,30,31].

The viscosity of polymer solutions arises from the hydration of polymer chains, where oxygen atoms form hydrogen bonds, resulting in irregular coils. As hydration continues, more water molecules get trapped within the expanded structure, further increasing viscosity [34]. An additional numerical suffix was introduced to indicate the viscosity of the polymer solution expressed in millipascal-seconds (mPa·s), which was strongly linked to the molecular weight of HPMC. The viscosity grade varies from 3 to 100,000 mPa·s and is measured in a 2% aqueous solution (*w*/*w*) at 20 °C. In addition to the numerical value, the letters “C” and “M” suggest a multiplication of 100 or 1000, respectively. In the case of hydrophilic matrix tablets, HPMC with a higher viscosity grade is used for highly soluble drugs, while lower viscosity grades are chosen for drugs with low solubility. Based on the API’s desired release mechanism, the formation of the gel layer is of great importance [35]. The degree of polymerization (DP) is closely linked to the polymer’s viscosity grade. In normal conditions, the increase in the number of monomers in the chain and, consequently, the molecular weight is proportional to the rise in the viscosity of HPMC solutions, with the process being more visible for lower DPs [36,37].

For labelling purposes, additional suffixes are also used for identifying the different characteristics of HPMC products, such as the following: “P” which identifies Methocel^®^ Premium grade products, “LV” which is used for low viscosity products, “CR” refers to controlled release products, and “DC” denotes HPMC variations used for direct compression, while “G”, “S”, and “FG” stand for granular, surface treated, and food grade abbreviations [38].

Concerning the availability of the polymer, hypromellose is highly used and easily accessible worldwide through different trademarks: Methocel^®^ (Dow Chemical Company, Midland, MI, USA), Metolose^®^, Pharmacoat^®^ (Shin-Etsu Chemical Company, Tokyo, Japan), Benecel^®^ (Ashland, Zwijndrecht, The Netherlands), and Mecellose and Anycoat (Lotte Fine Chemical, Ulsan, Republic of Korea). In terms of comparison, each subgrade provides distinct characteristics and therefore, different applications within the pharmaceutical field, as highlighted in Table 2. Recently, a new grade of HPMC was developed as a solution to poorly soluble drugs [32,39,40,41,42]. Affinisol (DuPont, Wilmington, DE, USA) presents improved solubility in organic solvents and can be used in thermally demanding technologies such as hot melt extrusion and spray-drying [27,43].

## 3. Alterations of HPMC Structure by Other Chemical Compounds

Since HPMC is a non-ionic molecule, the interaction risk is reduced; as a result, in the following chapter of this review, they will be called as alterations. Some alterations were noticed in the case of hypromellose with different chemical ingredients that, in some cases, can also serve as excipients, a fact that increases the importance of verifying the already published available data regarding the compatibility studies for both excipient–excipient and excipient–active ingredients. Even though the excipients are considered chemicals that are inert and compatible with most of the active ingredients, there are several cases where an HPMC structure was altered by the presence of other excipients.

### 3.1. Alterations Produced by Ionic Salt

Almeida et al. conducted a study where low molecular HPMC was used (Methocel E5 LV), and the presence of different monovalent and bivalent salts and thermogelation temperature were studied. During the DSC study, it was noticed that salt concentration (KCl, NaCl, and CaCl_2_) tended to decrease t_onset_ and the endothermic peak surface area. In conclusion, gelation properties are influenced by the presence and concentration of salts [44]. This fact can lead to changes in viscosity and gelation behaviour.

### 3.2. Chemical Interaction with Surfactants

The interactions of (HPMC) with the following cationic surfactants is explored below:

- Ethane-1,2-diyl bis (N, N-dimethyl-N-hexadecylammoniumacetoxy) dichloride.
- Pentanediyl-1,5-bis dimethylcetylammonium bromide.
- Hexanediyl-1,6-bis dimethylcetylammonium bromide.
- Cetyltrimethylammonium bromide, a conventional surfactant, has been studied employing surface tension and rheology evaluation [45].

A stronger HPMC–surfactant interaction was noticed in the case of the first three previously mentioned surfactants compared to the conventional surfactant, as indicated by the values of the critical aggregation concentration and critical micelle concentration. The HPMC–surfactant interaction produced polymer–surfactant micelles, and it was underlined that by increasing the hypromellose amount, the critical aggregation concentration and critical micelle concentration of the surfactant increased [45].

Kraisit et al. developed HPMC K4M-based orodispersible films (ODFs) where furosemide was incorporated in the polymeric mixture as a nanolipid carrier using the solvent-casting technique and applying a Box–Behnken factorial design. The authors have stated that by increasing the polymer concentration and by decreasing the surfactant (Cremophor^®^ RH 40) concentration, the tensile strength improves, whereas by decreasing the film-forming agents’ amount and increasing the Cremophor^®^ RH 40 concentration, the elongation is improved, implying a better elasticity of the film [46].

Sodium lauryl sulphate is a chemical compound that can be added to the dissolution media for active ingredients that exhibit low solubility in water (BCS class II and IV). Rede et al. evaluated diclofenac sodium HPMC-based tablets (Metolose 90SH-4000SR—HPMC type). The release of the selected API was evaluated at different concentrations of sodium lauryl sulphate, noticing that the amount of sodium diclofenac released increased with the sodium lauryl sulphate increase, with an exception being noticed below 0.03% pH = 4, (lower than the critical micellar concentration), where the amount of API decreased due to the swelling phenomenon [47].

### 3.3. Incompatibility with Strong Acids and Bases

Usually, the pH of an HPMC solution varies between 6.47 and 7.87, so it can be described as slightly neutral [48]. Punitha et al. evaluated the influence of pH on a 0.4% solution of HPMC in a pH range between 4.2 and 9.2, noticing low-velocity values at acidic pHs due to the coiling nature of HPMC, and the fact that an HPMC–water interaction took place instead of the polymer–polymer interactions that were observed at alkaline pHs, where the HPMC molecules extended and polymer–polymer interactions were noticed [49]. In conclusion, very low pHs and increased alkaline pHs (>9.2) can produce alkaline hydrolysis of the cellulose backbone, disrupting the polymer chains and decreasing the viscosity of the mixture. In a strongly alkaline medium, HPMC can undergo a deprotonation of hydroxyl groups, modifying its solubility and gelation properties.

At very low pHs (pH < 3), the protonation of hydroxyl groups can also alter the solution’s viscosity and the polymer’s viscosity under extremely low pH conditions, causing precipitation or phase separation [50,51].

### 3.4. Interaction with Oxidizing Agents

An experiment where Zupanc and collaborators evaluated HPMC degradation was conducted on 1 and 2 g/1000 mL HPMC solutions, which were treated with H_2_O_2_ at different temperatures (25 and 60 °C) for 30 min [51]. HPMC’s molecular weight initially decreased by 12–14% at 25 °C, while treating the macromolecule solution with H_2_O_2_ decreased it by 45–47% at 60 °C. As a result, higher temperatures are producing the oxidative cleavage of the β-1,4-glycosidic bonds (induced by H_2_O_2_). Similar behaviours were noticed in the case of other important macromolecular ingredients (hyaluronic acid), where, due to oxidative degradation, molecular mass decreased with a higher percentage at higher temperatures compared to room temperature [51].

## 4. HPMC Applications in Pharmaceutical Technology

HPMC plays a crucial role as a multifunctional excipient in the pharmaceutical industry. It is commonly used as a binder in tablet formulations, ensuring structural integrity and durability in controlled-release dosage forms and enabling sustained drug release over time. Its film-forming properties make it an ideal choice for coating tablets, providing a protective barrier that enhances stability, masks taste, and facilitates swallowing. Additionally, it can be used in capsule manufacturing as a gelling agent. Its utilities in pharmaceutical technology will be further summarised, and the chosen main roles of the film-forming agent and coating agent will be detailed in the following chapters.

### 4.1. Binding Properties

Due to their excellent adhesive properties, binders are commonly used to produce solid dosage forms and enhance the mechanical strength of granules or tablets. They help hold the ingredients of a tablet together, ensuring integrity during manufacturing, storage, and transportation [52]. HPMC is considered a versatile binding agent as it is compatible with both soluble and insoluble drugs. The viscosity of a binder is a crucial parameter to consider during granulation, as it directly influences the strength of the resulting granules [53]. The lower viscosity grades of HPMC serve as both a binder and a disintegrant in tablets, pills, and granulations, whereas higher viscosity grades function solely as binders. Concentrations ranging from 2% to 5% *w*/*w* can be used as a binder in either wet or dry granulation processes, depending on the specific types and requirements [54].

For example, Fristiohady et al. conducted a study to evaluate the effectiveness of HPMC as a binder in tablet formulations with *Cassia siamea* extract, observing the effect of HPMC and examining the impact of varying HPMC concentrations (2%, 3%, and 4%) on the physical properties of the tablets. The tablets were evaluated in terms of weight, thickness, hardness, and disintegration time. All physical quality standards were met except for that of tablet hardness, which proved to be greater than expected. This may be attributed to the high concentrations of lubricants and particles interlocking and undergoing plastic deformation facilitated by the binder [55].

### 4.2. Film-Coating Material

Among cellulose derivatives, HPMC is a polysaccharide widely used for coating formulations due to its “film-forming, transparency, flexibility **, ** and stability properties” [56]. These attributes are responsible for forming tough and flexible films that protect fragile tablets from environmental factors (light, oxygen, pH, moisture) and resist abrasion. Moreover, the resulting film masks the unpleasant taste or smell of the drug and improves the appearance of the tablet [57]. Taste masking has become a potential tool in the pharmaceutical industry to improve patient compliance and the marketing success of a product.

To optimize production efficiency and reduce coating time, the lowest possible viscosity grade of the polymer is generally preferred, as it allows for a higher solid content in the coating solution with a lesser amount of water. The apparent viscosity of an aqueous HPMC solution is directly related to the molecular weight of the HPMC polymer, with a reduction in molecular weight leading to diminished physical properties of the film coat [58]. The higherviscosity grades of HPMC provide films with good tensile strength, but their films have poor adhesion to the core surface and can easily peel off the tablet surface [59]. Generally, HPMC is used in concentrations of 2–20% *w*/*w* in film-forming solutions for coating tablets [54].

In another study, Iswandana et al. studied the film-coating capacity of HPMC by formulating and developing film-coated tablets containing *Momordica charantia.* The tablets were prepared by wet granulation using carboxymethyl cellulose as a binder and then coated with HPMC 5%. The purpose of the study was to improve the physical appearance and mask the unpleasant taste of a bitter melon. The results complied with the criteria of a good physical appearance and lower bitterness levels [60]. In another study provided by Abbaspour et al., HPMC was used as a film-coating agent. The study aimed to design and evaluate a moisture-resistant film formulation based on HPMC and microcrystalline cellulose and compare it with Sepifilm^®^ as a commercial gastro-soluble composition for the film coating of moisture-sensitive solid dosage forms (aspirin). The formulations were assessed in terms of mechanical strength, moisture permeability, and morphological properties. It was concluded that HPMC films could be applied as a moisture-resistant film coating, providing acceptable stability for aspirin tablets [61].

### 4.3. Biological Adhesive

Hydroxypropyl methylcellulose plays an important role in the field of mucoadhesive drug delivery systems, finding applications across numerous therapeutic domains. HPMC is extensively used in controlled-release formulations due to its thickening, gelling, and swelling properties, forming clear, stable, and odourless hydrogels. Moreover, it enhances contact with the mucous membrane and facilitates slow drug release, achieving the purpose of the treatment [62]. Its bioadhesive properties are attributed to the presence of the hydroxyl functional groups in HPMC molecules that can form hydrogen bonds with water and other polymers. This facilitates the absorption of large quantities of water, followed by swelling as a result of their hydrophilic nature. Furthermore, hydrogen bonding interactions between polymer chains and the mucin glycoproteins produced by epithelial tissues contribute to the mucoadhesive strength of HPMC [16,63]. As a mucoadhesive excipient, HPMC can be strategically utilized on the mucosal linings of the oral cavity and gastrointestinal tract, effectively exhibiting its strong mucoadhesive properties [64].

Another research group led by Peh et al. have investigated the suitability of SCMC (sodium carboxymethyl cellulose) and HPMC K4M films as drug delivery systems for buccal delivery. The mechanical properties and in vitro/in vivo bioadhesive strength capacity were evaluated, with HPMC films showing greater in vivo bioadhesion, although their in vitro bioadhesive strength was lower than that of the SCMC films. The authors concluded that HPMC films might be preferred over SCMC films as drug carriers for buccal delivery due to their superior toughness, elasticity, bioadhesiveness in vivo, and a more controlled swelling behaviour in the oral cavity [65]. Araujo et al. developed a microemulsion gel obtained from hydroxypropyl methylcellulose films for the transdermal administration of Zidovudine. They observed that the addition of HPMC 1.2% to the film formulation caused an increase in viscosity, ensuring that the gel remains at the site of application for a prolonged time, enabling a transdermal permeation of the active ingredient [66].

### 4.4. Gelling Agent

Hydroxypropyl methylcellulose can also be used as a gelling agent in the production of cosmetics and medicines since it can produce a clear gel, dissolve easily in water, and has low toxicity. Additionally, HPMC forms a neutral, clear, and colourless gel that remains stable within a pH range of 3–11, offering strong resistance to microbial growth and enhancing film strength upon drying on the skin. The results of previous research indicated that HPMC bases had a good drug release rate and wide spreadability [67,68]. As a gelling agent, HPMC showed the most optimal physical stability in gel preparations compared to Carbopol. Its advantages include good skin dispersion, a cooling effect, no clogging of skin pores, and easy water-washing. Nevertheless, it is utilised in the pharmaceutical industry because it exhibits good stability under different conditions. Even when exposed to heat and humid conditions, HPMC gels maintain their homogeneity, pH, clarity, texture profile, and rheological properties without significant changes [69].

The studies showed that HPMC is mostly used as a gelling agent in concentrations ranging from 1% to 4%. Tanwar et al. have developed topical gel formulations with Diclofenac sodium using different gelling agents (HPMC K4M, Carbopol 934, carboxymethylcellulose sodium salt medium viscosity 200–400 cPs, and sodium alginate) at various concentrations. They used 1%, 1.5%, and 2% HPMC and concluded that a high polymer concentration increases viscosity, which leads to a consequent decrease in drug release [70]. Daood et al. have used 1%, 2%, and 3% HPMC for the preparation of a metronidazole topical gel, while Nursal et al. have developed an emulgel with 1–4% HPMC concentrations. All formulations showed acceptable physical properties concerning colour, homogeneity, consistency, spreadability, and pH value [71,72].

### 4.5. Encapsulation Material

Hypromellose, a widely accepted cellulose-derived material, has demonstrated its versatility as a polymer with easily adjustable solution properties, making it suitable for producing hard two-piece capsules using standard manufacturing equipment [73]. HPMC capsules have presented an attractive alternative to gelatine capsules in recent years as a result of their promising properties. The main limitation of hard capsules resulted from an exchange of moisture between the capsule shell and the fill. Their usefulness is related to their capacity to protect the contents in the presence of moisture. The moisture content of gelatine capsules may vary between 13% and 16% according to the weight of water, compared to HPMC capsules, where they can remain stable from 2% to 6% [74,75]. Variations in moisture content may influence the properties of capsules, such as stability, crystallinity, or efficiency. Yang et al. have proven that HPMC capsules, in comparison to gelatine capsules, can more effectively protect high, moderate, and low hygroscopic contents from outside moisture absorption, showing weaker moisture sorption [76]. Moreover, hypromellose capsules require unique manufacturing methods because hypromellose solutions do not naturally undergo a self-solution-gel transition without the use of process aids, leading to variability in the quality properties [77]. Some of their advantages are as follows:

- They are semi-synthetic in nature, derived from plant cellulose.
- No cross-linking issues.
- They are easier to swallow.
- They form a flexible film.
- Water does not act as a plasticiser for HPMC, exhibiting enhanced stability in various conditions (temperature, humidity).
- Their non-ionic nature makes them compatible with most of the commonly used excipients as well as APIs.
- They are preservative-free, allergen-free, starch-free, and gluten-free.
- The adhesion properties and optimal shell texture of HPMC films enable the application of a modified-release uniform coating.
- They do not require Transmissible Spongiform Encephalopathy certification (as in the case of animal-derived gelatine capsules) [78,79].

### 4.6. Suspending Agent

The higher viscosity grades of HPMC can be used as suspending agents and suspension-type liquid formulations in concentrations of 0.2–1.5% *w*/*w*. Their effect is satisfactory, being that it easily spreads, and the flocculation grain is smooth [80,81]. Although it can be used for this purpose, studies conducted by Tagliari et al. and Murthy et al. showed inferior results compared to other suspending agents, such as sodium carboxymethylcellulose or methylcellulose, in terms of the instability index, sedimentation velocity, and resuspendability of the suspensions [82,83].

### 4.7. Thickening Agent

The incorporation of HPMC into liquid systems enhances viscosity due to increased intermolecular friction among the solvated HPMC chains. Increasing viscosity helps keep colloidal dispersions stable because it delays particle aggregation. As a result, HPMC is used as a thickener and stabiliser in the pharmaceutical industry. The increase of the polymer’s average molecular weight at a certain temperature leads to a higher viscosity [21]. By exceeding a certain molecular weight, the chains tend to entangle, forming gels. In a study conducted by Stolic-Jovanović et al., HPMC was used as a thickening agent at a concentration of 1% [84]. As a thickening agent, HPMC is used and commonly employed in ocular drug delivery systems. El-Kamel developed and evaluated a gel-forming solution to improve the bioavailability and decrease the side effects of timolol. For this purpose, the rheological behaviour of various agents was studied, with HPMC 80–120 cP 2% exhibiting a high viscosity [85].

### 4.8. Controlled-Release Polymer

Hydroxypropyl methylcellulose is a commonly used release-controlling polymer in hydrophilic matrix tablets. The principle of the polymer’s controlled-release property is that, upon contact with a dissolution medium, the polymer found on the tablet’s surface rapidly hydrates, forming a gel layer. The gel layer acts as a physical barrier, protecting the tablet core and controlling the drug release from the matrix. Drug release usually occurs by two routes: a diffusion of molecules through the gel layer and a gradual degradation of the polymer matrix at the interface with the dissolution medium [86]. Several researchers reported that molecules that are highly soluble in water follow a diffusion release mechanism, whereas poorly water-soluble molecules are mainly released by an erosion mechanism [14]. The release rate is correlated to the porosity and tortuosity of the pores or channel networks, both of which are attributed to the polymer’s swellability. Variables such as particle size, viscosity, and concentration of HPMC can also influence drug release from the matrix. An increase in HPMC particle size generally leads to higher drug release rates from matrix tablets, while higher viscosity grades often result in slower release rates [87]. The variety of HPMC grades with different substitution degrees and viscosities makes it a versatile matrix for the controlled release of a wide range of drugs with varying solubilities and doses [88].

Pani et Nath studied the controlled release of nateglinide from HPMC-coated tablets using a 3^2^-randomized full factorial design. They obtained an optimal formulation containing HPMC K15M 5% and K100M 15%, which showed promising statistical results (the maximum value of the similarity factor and minimum value of the difference factor) and the desired stability during accelerated stability tests. The projected release of nateglinide from the controlled-release tablets was 26.63% *w*/*w* at 1 h, 33.30% at 2 h, 39.97% at 3 h, 46.64% at 4 h, 59.98% at 6 h, 73.32% at 8 h, 86.66% at 10 h, and 100% at 12 h. This sequence of values was taken as the reference release profile for comparison with the test formulation. The optimal formulation presented the most similar results to the theoretical drug release profile [89]. Ohara et al. carried out a study to analyse the release mechanism of indomethacin, a poorly water-soluble drug, from matrix systems composed of ethyl cellulose (EC) and hydroxypropyl methylcellulose HPMC, TC-5EW in a 1:1 (*w*/*w*) ratio. The results revealed that the drug dissolution mechanism was influenced by the structural characteristics of the polymeric matrices, which were, instead, affected by the preparation method and the pH of the dissolution medium. As the pH of the dissolution medium was lowered, the dissolution rates markedly decreased. At pH 3.5, roughly 80% of the HPMC diffused out within 6 h; yet, only about 20% of indomethacin (IND) was released. This is attributable to the hydrophobic affinity between ethyl cellulose and IND: in acidic conditions, once HPMC departs from the solid-dispersion granules, IND remains adsorbed onto the ethyl cellulose matrix instead of entering the bulk dissolution medium, and it is released only gradually thereafter. It was implied that the hydrophobic interaction between indomethacin and ethyl cellulose occurred in the lower pH region and strongly delayed the dissolution of IND [90].

## 5. HPMC as a Key Excipient in the Development of Coated Tablets

Tablets are one of the most prescribed pharmaceutical formulations. Due to some disadvantages that the active ingredient might produce (unpleasant taste or mouthfeel), some tablets are coated with sugar or a polymeric film. In recent years, due to the disadvantages regarding the average mass that increased by almost 50% during sugar coating compared to a maximum 10% increase of the previously mentioned parameter, while applying polymer coating, the latter method tends to be used more often [59].

The hydroxypropyl group, the substitution degree, the heterogeneity, and the polymeric film permeability to water affect the APIs’ dissolution profile. The contact between water and the polymer produces HPMC hydration, producing a viscous gel that alters HPMC functionality. The hydrogen bonds formed between the oxygen atoms of HPMC (from the ether group), and H_2_O lead to polymer extension. The hydrogen bonds produce a coil-shaped structure that tends to form more hydrogen bonds and entrap water, increasing the flow resistance [91]. HPMC is one of the polymers that can be easily used for polymer coating due to the following advantages:

- It produces an aqueous polymeric film.
- It is an easily processable excipient.
- It produces a transparent, resistant, and flexible film that protects the core.
- It upgrades the tablet’s appearance by improving the shininess and providing a homogenous aspect that can be easily evaluated macroscopically.
- It improves the organoleptic properties (taste and smell).
- It provides protection of the API from the action of environmental factors (air, O_2_, temperature).
- It avoids incompatibilities by incorporating one ingredient in the core and one in the coating polymeric mixture.
- It facilitates the easy identification of the pharmaceutical product, improving administration efficacy.
- It has improved flowability, which is desired in the pharmaceutical industry to enhance the production yield.
- It has resistance to external factors such as abrasion [92].

In most cases, low viscosities of HPMC produce films with decreased tensile strength, while high-viscosity HPMC produces films with improved tensile strength but an increased risk of peel-off. As a result, when high-viscosity HPMC is used, another polymer needs to be considered [91]. Therefore, an HPMC–Hydroxypropyl cellulose (HPC) combination is utilised, with the latter improving film adhesion.

Another parameter that might influence drug release in the case of HPMC-coated tablets is particle size, noticing that small particle size produces stronger tablets due to interparticle cohesiveness, whilst larger particles enhance dissolution since the space around each particle is not fully occupied, producing spaces where the water can penetrate [92].

The main factors that influence the dissolution profile of an API that can be found in the core of a coated tablet are as follows:

- Disintegration ability.
- Diffusion of water through the pores.
- A diffusion and erosion combination for the different particle sizes of HPMC.
- Drug/HPMC ratio.
- Drug solubility.
- Compression force.
- HPMC viscosity grade.

Other different methods can be applied to obtain variation regarding the dissolution profile; for example, the coating layer that contains a hydrophilic polymer could incorporate a small amount of API that can produce a fast therapeutic effect and another amount that could be incorporated in the core, where different binders can be used to delay the active ingredient release, combining two different methods of active ingredient release. The amount included in the polymeric layers has to be therapeutically effective in minimal doses since the substances incorporated in polymeric matrices underscore a crystalline structure, producing mechanical film alteration. Also, there will be an increased possibility of “film-peel-off”.

The coating with active ingredients is increasing some issues since the active ingredients can be soluble or insoluble in the aqueous coating solution; in the first case, the active ingredient dissolves in the polymeric mixture, while in the second case, the API is suspended. As a result, in the latter case, the risk of discontinuities in the coating film needs to be considered. To reduce the influence of insoluble active ingredients in the polymeric–coating mixture, the particle size needs to be considered and evaluated during the whole process (to remain constant) to decrease the risk of clogging and film-related problems. Since the coatings are obtained by the spray-drying process, the following variables need to be taken into consideration:

- Spraying rate.
- Inlet air temperature.
- Residual moisture.
- Pan speed.
- Atomization process.
- Drug properties [93].

To achieve a successful formulation, a compatibility study between the API and the excipients must be conducted before the coating formulation composition starts. If two active ingredients are not compatible with each other, one can be included in the core and the other one in the coating film, and they can be separated by a functional layer. After this step takes place, the preparation can start, and the coating mixture can be developed, respecting a specific order and a specific range of temperature depending on the polymer type [94].

Kim et al. developed fixed-dose tablets using three different layers: the first one where metformin was included in an extended-release core, an inert mid-layer, and an external layer with an immediate release that contained glimepiride. The intermediate layer was necessary since it was concluded that the inert mid-layer was necessary to reduce the risk of delaying the release of glimepiride by the metformin core. In this case, the HPMC with 100 cP was used in the core to delay the release of metformin, while lower viscosity HPMC (4.5 or 6 cP) was used to obtain the suspension that was sprayed over the intermediate layer [95].

**Figure 2:**
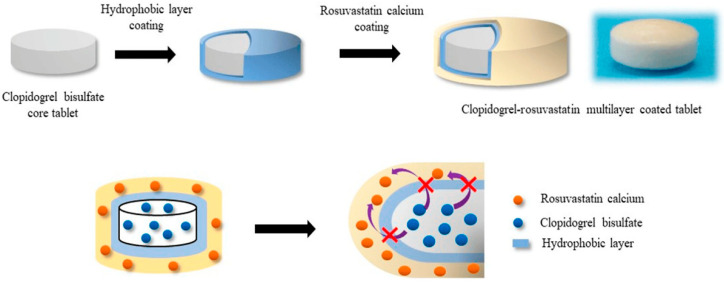
The rosuvastatin and clopidogrel multi-layered tablet after Seo and Han [96].

Hypromellose has also been considered in another study as a coating agent alongside carnauba wax, sodium stearyl fumarate, and glyceryl behenate, where a combination of rosuvastatin and clopidogrel was considered as the active ingredient. By combining these two ingredients, an increased risk of incompatibility has to be considered; as a consequence, the two APIs were included in different parts of the coated tablet, with clopidogrel in the core, while rosuvastatin was incorporated in the coating–polymeric mixture. Compatibility was underscored by the amount of lactone assayed through an HPLC method, showing that, in this case scenario, the hydrophobic glyceryl behenate was the best solution, inhibiting lactone production (<0.02%) compared to the other three selected excipients that displayed a lactone production in the range of (0.16–0.25%) [96]. The structure of the multi-layered coated tablet that contained both rosuvastatin and clopidogrel is highlighted in Figure 2.

## 6. Hypromellose as a Film-Forming Agent

Hypromellose is one of the most used film-forming agents, being compatible with all the preparation methods used to develop polymeric films with different utilities (fast-delivery—orodispersible films and local/systemic delivery—mucoadhesive films, depending on the active ingredient effect and properties and the formulation itself) [3,5,16]. Since it is compatible with various active ingredients and excipients, and also because it can be present at different polymerization grades and viscosities, this ingredient has been extensively used in the development of polymeric films, usually with its concentration or viscosity being the parameters that were varied to determine which is the most useful type of HPMC in specific formulations. Generally, the type of polymer, the viscosity of the polymer, and its concentration are the parameters that produce varieties regarding the evaluated parameters (average weight/mass, thickness, folding endurance, tensile strength, disintegration time, dissolution test, etc.) [36,37,38].

For example, in the case of the average weight, by increasing the concentration of the polymer, the mass tends to increase, a fact that can also be noticed by increasing the viscosity of the polymer. Generally, it is expected that an increase in the polymerization degree will produce films with a higher mass. For example, if the same amounts of HPMC E3, HPMCE5, HPMC E15, and HPMC E50 are used and the other ingredient amounts are not varied, there is a high possibility that the films’ average mass increases by following the viscosities of the aqueous solution of the polymers (3 mPa for a 2% HPMC E3, 5 mPa for a 2% HPMC E5, 15 mPa for a 2% HPMC E15 solution, and 50 mPa for a 2% HPMC E50 solution). Depending on the other ingredients’ amounts and influence on the average mass, this stipulation can be applied in most cases when polymeric films are developed. In some cases, the interaction between the inputs needs to be considered as a result, Quality by Design needs to be applied to better outline the mutual effect of the factors [27,36].

Thickness is influenced in most cases by HPMC concentration and type, usually, through an increased concentration of the polymer producing a higher thickness and by using the same amount of HPMC but with different polymerization degrees; due to the different viscosities of the mixture obtained, different thicknesses will be obtained respecting the following order HPMC E50 > HPMC E15 > HPMC E5 > HPMC E3 (in most of the cases). As mentioned before, this behaviour will be noticed in the case of only varying this parameter; if other parameters are varied, a one factor at a time approach or a more modern one such as quality by design (QbD) (if the factors are varied simultaneously), should be applied, where, in addition to the factors themselves, the interaction between the factors and the way that they are influencing the dependent parameters is underscored [36].

The mechanical properties are also influenced by polymer concentration, since this ingredient provides a better matrix structure. Frequently, a decrease in the polymer concentration provides lower mechanical properties, such as a reduced fold number or reduced tensile strength, that can be improved by increasing the polymer concentration. Another factor that can influence the mechanical properties, is the amount of API that will be included in the polymeric matrix, usually an increased amount of the crystalline ingredient produces lower mechanical properties; normally, the thin films tend to be less resistant and can only resist one or a few folds, a fact that implies a packing method correlated with this mechanical inconvenience for better storage of the final product until the patient starts using it [97,98].

The disintegration time is influenced by the hypromellose type, viscosity and concentration [1,2,3]. In the case of orodispersible films, a high concentration of the polymer can outline an increased disintegration time by deprecating the limit admitted in the case of orodispersible tablets (30 s USP 45 and 180 s Ph. Eur. 11), with a special mention that in most cases it is expected that the films will disintegrate within seconds to obtain a fast release of the API [29,99]. To improve this parameter, a lower concentration of a film-forming agent needs to be used, but a compromise needs to be made between the mechanical properties that are supported by an increased amount of the film-forming agent and the disintegration time, where, usually, by increasing the HPMC concentration, the parameter can deprecate, exceeding the in-force pharmacopeial stipulated maximum limits [29,100].

Normally, if the API is dissolved in the polymeric matrix, the disintegration time can be considered a preamble for the dissolution test, whilst if the active ingredient is suspended in the polymeric matrix, the dissolution profile might not be strictly correlated with the disintegration time [101]. Also, in the case of BCS II and BCS IV active ingredients, even if the polymer might improve the amount of active ingredient released at different time-frames, the fact that the APIs from these two categories have low hydrophilicity might imply challenges regarding the composition of the dissolution media, the amount of dissolution media used, and the in vitro–in vivo results correlation [59]. Normally, the use of HPMC E50 instead of the lower viscosity varieties of HPMC produces a decreased amount of API released or an extended release of the API which is not desired in the case of ODFs but can represent an advantage in the case of mucoadhesive films where a different type of release is expected, usually either an extended or a modified release [56,86].

Usually, by preparing the polymeric films through the solvent casting method, good mechanical properties and uniform thickness are achieved, but the disintegration time might vary due to the drying process, which has to be controlled to avoid solvent retention [98,102,103]. In this case, the use of disintegrants might be a solution, but the inclusion of disintegrants in the composition might cause discontinuities and degraded mechanical properties. Besides, the macroscopic properties of the film might be seriously damaged [100].

Hot-melt extrusion (HME) is a method where the polymer is melted and mixed with the other selected ingredients, followed by an extrusion into thin polymeric films [27]. The film disintegration time obtained through this method tends to be shorter in comparison with the previously mentioned method; even so, some special mentions need to be considered:

- The use of low molecular polymers (HPMC E3 instead of HPMC E50).
- The use of surfactants (usually, the disintegration time tends to improve by using hydrophilic surfactants such as Tween 80, but palatability needs to be considered, and the taste needs to be improved while using this type of excipient; Tween might have a bitter aftertaste).
- It is a challenging method when applied to thermosensitive APIs [27,43].

The ODFs developed through other methods, such as 3D printing or the electrospinning method, tend to underscore disintegration times within seconds, implying improvements regarding the dissolution profiles, and representing a huge advantage [97,98,101]. This can be applied especially in the case of active ingredients that highlight low solubility, and since most of the newly discovered APIs belong to the 2nd or 4th BCS, the incorporation of an API in an amorphous matrix improves hydrophilicity and also the amount of active ingredient released at different time-frames. However, these two methods have disadvantages. For example, electrospinning is a complex method not usually used in large-scale manufacturing. At the same time, in the case of 3D printing, the disintegration time might vary depending on the materials and the settings used for the 3D printer [8,97].

Since many ingredients tend to have issues with hydrophilicity, in most cases, the problem is solved by developing solid dispersions (SDs). Even though in the case of ODFs, SDs are rarely used, the proposed pharmaceutical formulation itself represents a way of improving water-solubility, since in many cases an amorphous dispersion of the active ingredient is noticed, providing better in vitro dissolution behaviour and improving also the in vivo release profile [90]. The transition of the API from a crystalline structure into an amorphous state can be noticed with the help of the following:

Differential Scanned Calorimetry (DSC): Usually, the specific endo/exothermic peak of an API disappears while the amorphous mixture is formed, or its intensity decreases dramatically, a fact that might imply an improvement in solubility, but in some cases some other processes can be noticed such as a lack of stability, as well as oxidation that can be outlined with the help of other analytical tools. In the case of HPMC or other polymers analysed by the means of DSC, water loss can be noticed before 100 °C, starting from 40 to 50 °C, so when a binary mixture of an API with a polymer is analysed, the characteristic peak of the API tends to be noticed if the API transitions from a crystalline structure into an amorphous state. Otherwise, the peak is shifted or disappears depending on the characteristics of the newly formed amorphous phase. For example, CBD, an API previously analysed in terms of calorimetry, showed a specific melting point at 69 °C, which was also noticed when a binary mixture of CBD with HPMC was analysed, with the peak showing a decrease in its intensity [104,105]. In the same study, an alteration of the endothermic peak of an API was noticed when the binary mixture between CBD and Poloxamer 407 was analysed, outlining the fact that an interaction between these two was noticed, probably a solubility improvement as shown in another study [106]. Usually, if the amount of crystalline ingredient is increased, the intensity of the peak decreases, whilst if the amount is low, the peak disappears [107].

FT-IR spectroscopy: Some specific bands are noticed which are characteristic of different chemical groups; in this case, even after amorphization takes place, some of them can still be noticed, but in many cases their intensity lowers or small shifting tends to be noticed since the bands of 5–10 different ingredients are interfering, depending on composition [107,108]. For example, for HPMC, the following bands were outlined: the first one at approximately 3458 cm⁻^1^ due to the stretching of the O-H bond, and other bands in the range of 2800 to 3000 cm⁻^1^ for C-H stretching. The band at 1047 cm⁻^1^ is specific to the C-O stretching vibration. Other specific bands of the HPMC are noticed at 1456 cm⁻^1^, attributed to C-H bending, and 1118 cm⁻^1^, which is characteristic of the C-O-C pyranose ring vibration. In most cases, if a binary mixture is analysed, the specific bands tend to shift for both the excipient and the API [105]. If a polymeric film is analysed with FT-IR, the bands tend to shift or disappear, or in some cases, lose their intensity, making the highlighting of specific bands for different chemical groups that are specific to the components extremely difficult.

XRPD: In the case of the active ingredient, available in crystalline form, 2θ angles are numerous and specific for each ingredient, even for the other excipients that are available in a crystalline form. For example, HPMC has only two specific 2θ angles at 8.4 and 20° which can be noticed in the case of the orodispersible films developed; small changes regarding the intensity being noticed, and their shape is different compared to the crystalline ingredients that tend to have multiple multidentate peaks [107]. By the means of XRPD, the crystalline or amorphous state of the excipient/formulation or binary mixture can be stated. Through this pharmaceutical formulation, an amorphization is noticed as a result of the specific 2θ angles either being less numerous and tending to have a lower intensity or disappearing, announcing that an amorphous mixture was formed [97].

A study conducted by Hussain et al. outlined the DSC thermograms and FT-IR spectrograms of three active ingredients (aspirin, nystatin, and hydrochloric lidocaine), two film-forming agents (HPMC E5 and polyvinylpyrrolidone K30—PVP-K30), and one agent belonging to the buccal film [107]. It was noticed that each active ingredient had a specific endothermic point, while in the case of the polymers, water loss could be observed with different t_onset_ and t_endset_. In the case of buccal films, the endothermic peaks of HPMC E5, PVP K30, and Lidocaine HCl merge, outlining one endothermic peak, while the endothermic characteristic peaks for aspirin, nystatin, and lidocaine hydrochloride disappear. A new peak at about 400 °C is formed close to the peak of nystatin, but since an amorphous phase is formed, it is pretty difficult to establish if the peak belongs to nystatin or if it is formed due to the interaction between the excipients and the active ingredients [107].

In the case of the FT-IR spectrograms, different specific bands can be noticed for the active ingredients, excipients, and buccal films, each one of them being characteristic of one specific bond. It was highlighted that the number of specific bands and their intensity tend to reduce, and some of them might vanish as a result of the amorphous mixture, but even so, some wavelengths for each of the three ingredients could be noticed in the polymeric mixture [107].

In the study conducted by Silva et al., the active ingredient (doxazosin mesylate), the film-forming agent (HPMC E6), and several orodispersible film formulations with or without a plasticizer were characterized, noticing that only in the case of the active ingredient were some sharpened specific 2θ angles noticed, while in the case of the ODFs the specific 2θ angles for the HPMC E6 were noticed [97].

Until now, different pharmaceutical ingredients have been selected as model drugs for incorporation in ODF matrices. As can be seen in Table 3, usually, the amount of API is lower than 100 mg; in many cases, the active ingredients produce a therapeutic effect in small amounts [97,98,102,103,108]. In many cases, the method selected to develop orodispersible films is the solvent casting method, followed by 3D printing, with the latter being chosen due to the fast disintegration times exhibited and very good releasing profiles [97,98,101,107,109]. In addition to pharmaceutical APIs, the incorporation of phytocomplexes in ODFs was also tried (for example, clove oil, which was previously nano-sized to improve the incorporation in the polymeric matrix) [110]. In all the cases considered in Table 3, a low-viscosity HPMC was considered as a film-forming agent, either alone or in combination with other polymers (HPC, maltodextrin, PVP-K30) [111,112,113,114,115]. By developing ODFs with two or more polymers, an improvement regarding the critical properties of ODFs might be noticed, since one of them might improve the mechanical properties while the other might enhance its disintegration ability.

Cross-linked hydroxypropyl methylcellulose (HPMC) has emerged as a versatile biomaterial in pharmaceutical and biomedical applications, with enhanced mechanical stability, controlled swelling behaviour, and sustained drug release profiles; it is employed in colon-targeted delivery systems, wound-healing films, injectable hydrogels, and bone tissue engineering scaffolds, through chemical cross-linking methods involving agents such as citric acid, PEG, or glutaraldehyde.

Various cross-linking strategies have been developed to tailor the properties of hydroxypropyl methylcellulose (HPMC) hydrogels for specific biomedical applications. Chemical cross-linking involves agents such as citric acid, glutaraldehyde, or polyethylene glycol.

## 7. HPMC Formulations Used in the Medical Field

**Figure 3:**
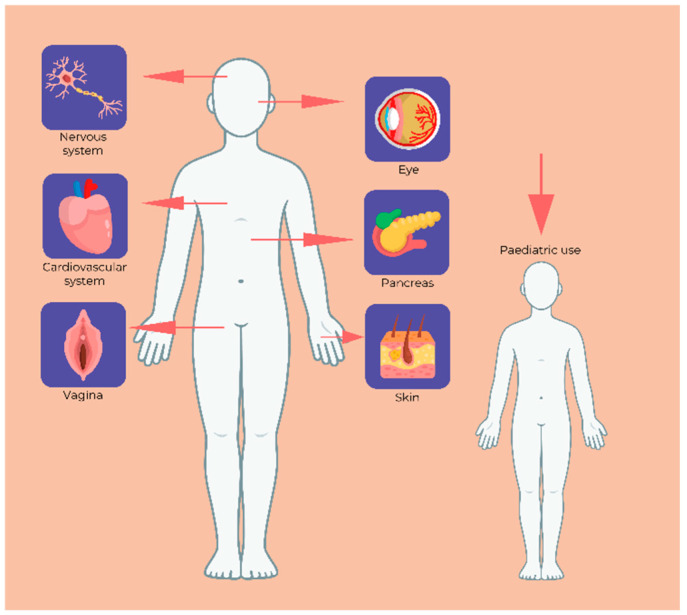
Applications of HPMC formulations.

HPMC dosage forms are widely used in various branches of medicine due to their biocompatibility, nontoxicity, and multifunctional properties, making them an essential component in medical treatments. The excipient can be incorporated in several pharmaceutical formulations, as previously underscored, that can be administered to treat several diseases located in different body compartments, as shown in Figure 3.

The latest research has shown that a variety of active ingredients for different conditions are being incorporated into HPMC formulations due to the polymer’s excellent properties. Some of them are listed below in Table 4.

The utility of the HPMC in the different pharmaceutical formulations used in different medical fields will be further underscored.

### 7.1. Biocompatibility

When choosing the right excipient, one of the most important aspects that must be considered is its biocompatibility. Hydroxypropyl methylcellulose is biocompatible because its chemically neutral cellulose backbone, modified only with small methyl and hydroxypropyl groups, leaves no reactive or charged sites that could injure tissue. Because its chemistry is close to that of natural cellulose, it lacks reactive or charged groups, resists metabolic activation, and has an extensive history of safe clinical and dietary exposure; HPMC reliably meets the practical definition of a biocompatible polymer. When used for oral administration, the polymer’s large, water-loving chains cannot cross healthy gut epithelium, so it is hardly absorbed and is excreted largely unchanged, minimizing systemic exposure. In a study, Jyoti et al. performed the cytocompatibility test of 0%, 2%, and 4% HPMC using Human fibroblast-like cells, derived from mouse fibroblasts. Based on the cytotoxicity results, a fairly acceptable viability of the cells was observed with 2% and 4% HPMC. The general survival rate of the cells for the 2% and 4% HPMC samples was satisfactory, at 74% and 81%, respectively, at 100% dilution [135].

### 7.2. Ophthalmology

In ophthalmic applications, HPMC can be used as a viscoelastic agent in eye drops, a gelling agent in injections, and a polymeric matrix in films, filaments, and inserts. The different therapeutic approaches are necessary due to the complex anatomical structure of the eye [21]. HPMC, as previously mentioned, can function as a thickener in fluid formulations due to its ability to hydrate easily, swell efficiently, and facilitate the controlled release of drugs, allowing its application either in topical treatment or intravitreal injection [136]. HPMC is considered to be one of the main ingredients in artificial tears. Liu et al. have conducted a study to develop a stable delivery system with increased bioavailability and enhanced corneal penetration of a hydrophobic drug such as tacrolimus. Hydroxypropyl methylcellulose 15 K was selected as the preferred stabiliser and viscosity-improving agent. They concluded that the formulations were promising ocular delivery systems, improving the efficacy of tacrolimus in anti-allograft rejection [137]. Esteban-Perez et al. have explored the potential of using a hybrid system (gelatine nanoparticles combined with HPMC 0.3%) for an effective ocular local administration of timolol maleate, a beta-adrenergic blocker used for glaucoma treatment [138]. Another study followed the design of a pH-triggered Carbopol 940 0.1%/HPMC F4M 0.1% in situ gel for the ocular delivery of dorzolamide hydrochloride. The optimal formulation was compared to other products existing on the market and showed better performance in intraocular pressure activity [139].

### 7.3. Diabetes

Another application of hydroxypropyl methylcellulose is in the formulation of sustained and controlled-release oral medications for diabetes. By forming a gel-like matrix in the gastrointestinal tract, HPMC ensures a gradual and consistent drug release, improving therapeutic efficacy and reducing dosing frequency, which enhances patient adherence. Sharma et al. have studied the effect of the different viscosity grades of HPMC on drug loading and the in vitro drug release of repaglinide with satisfactory results [140]. Moreover, a novel way of administering insulin to type 1 diabetes patients was developed in the form of a buccal mucosa adhesive film. A double-layer film was obtained using a combination of HPMC E50, 40–60 mPa·s 0.2% and polyacrylic acid 0.2% (molecular weight 5 kDa, 25 kDa, 90 kDa, 450 kDa, and 3000 kDa) as a mucoadhesive coating and ethyl cellulose N22, 18–24 mPa·s as a waterproof layer, ensuring the APIs’ controlled release. The therapeutic effect of the formulation was tested on type 1 diabetes rats with post-oral glucose administration; the films effectively stabilized blood glucose levels and maintained lower glucose levels for approximately 8 hrs. This formulation has proven to be a promising solution to circumvent the issue of poor medication compliance in patients taking pre-prandial insulin [141].

### 7.4. Neurology

Furthermore, HPMC is used in the formulation of sustained and controlled-release drugs for treating neurological conditions such as Parkinson’s disease, epilepsy, and Alzheimer’s disease. Due to the gel-like matrix, HPMC can enable the gradual release of neuroactive ingredients, reducing fluctuations in drug levels and improving therapeutic outcomes where consistent plasma concentrations are critical. Fathi et al. aimed to develop an intranasal gel with agomelatine, a melatoninergic antidepressant drug, for enhanced treatment compliance. To facilitate the permeation of the API through the mucosa, they used HPMC 4KM 0.2% as a mucoadhesive polymer, ensuring prolonged contact at the application site. The polymer was chosen depending on the gelation temperature upon the addition of different mucoadhesive polymers (sodium alginate, HPMC, Xanthan gum, Carbopol, sodium carboxymethylcellulose), with HPMC being chosen due to an acceptable gelation temperature for intranasal administration [142]. Moreover, scientists have tried to incorporate neuroactive substances in various types of HPMC pharmaceutical formulations, such as in situ gels loaded with risperidone or aripiprazole mucoadhesive nanoemulsions, to study the pharmacokinetic profile of the APIs [143,144].

### 7.5. Gynaecology

In gynaecology, HPMC is used in vaginal gels, suppositories, and bioadhesive films for the controlled release of medications. In a recent study, voriconazole cyclodextrin-based in situ gels were prepared using poloxamers and various mucoadhesive polymers. The optimal formulation containing 0.4% HPMC (Methocel E50 LV) demonstrated excellent gelling ability, gelling temperature, shear thinning behaviour, excellent mucoadhesion, and high drug uptake [145]. Another study found that tioconazole films obtained with chitosan and hydroxypropyl methylcellulose K4M were more active than pure drugs or traditional ovules and did not produce substantial haemolytic and cytotoxic effects, making them promising alternative dosage forms for the treatment of vaginal candidiasis [146]. Moreover, HPMC (400 cP) was used as a viscosity modifier to develop vagina suppositories containing prostaglandin P2 for the initiation of labour and cervical ripening [147]. A study conducted by Notario-Perez et al. reported that the combination of HPMC K100M and chitosan in the same formulation could be beneficial in preventing the sexual transmission of HIV. This is due to the ability of the resulting tablets to adhere to vaginal mucosa for up to 96 h, ensuring a sustained drug release over 72 h [148].

### 7.6. Paediatric Therapy

Another advantage of HPMC is that it is safe to use in formulations destined for paediatric administration. Choosing excipients with optimal safety, tolerability, and suitable physicochemical properties is crucial in developing paediatric formulations. A study aimed to prepare a nicardipine hydrochloride oral solution for the treatment of hypertension in children using carboxymethylcellulose and hydroxypropyl methylcellulose 4000 in different concentrations. The solution using CMC 0.1% and 0.5% was opalescent with visible aggregates of nicardipine on the magnet bar; thus, it was not suitable for further testing. The formulations containing HPMC 0.5% and 0.25% were opalescent, and nicardipine was evenly dispersed. The results showed that the solution with HPMC 0.5% was too viscous, in contrast to the concentration of HPMC 0.25%, whose viscosity seemed more appropriate for oral use. The formulation using HPMC 0.25% displayed good palatability and appropriate viscosity [149]. HPMC can be used to obtain gummy drug formulations that paediatric patients highly accept due to their pleasant taste, gum-like texture, interesting shapes, and colours. For this purpose, Tagami et al. incorporated lamotrigine, an antiepileptic drug, into gummy formulations composed of gelatine, HPMC 4000, syrup, and water. They observed that by incorporating the polymer, viscosity increased with little effect on the gummies’ hardness [150].

### 7.7. Dermatology and Dermato-Cosmetic Products

Hydroxypropyl methylcellulose plays a significant role in dermatology, particularly in topical formulations, wound healing, and cosmetic applications. Its biocompatibility, film-forming ability, and hydrophilic nature make it an essential ingredient in various dermatological products. In the development of an in situ spray for the delivery of an antibacterial drug for the treatment of hidradenitis suppurativa, it was found that the optimal concentration of HPMC K4M is 0.2%, showing excellent bioadhesion at the site of application [151]. A study developed and evaluated a film-forming gel of 5-fluorouracil (5FU) using different ratios of hydroxypropyl methylcellulose and zein for treating vitiligo. The results suggested that an increase in HPMC K4M concentration (2–4%) was associated with an enhanced release of 5FU, while a higher Zein concentration (1–2%) led to a decrease in 5FU release [152].

In dermato-cosmetic products, hydroxypropyl methylcellulose (HPMC) serves several critical functional roles that enhance both formulation stability and therapeutic performance. As a thickening agent and emulsion stabiliser, HPMC ensures a uniform, creamy or gel-like texture suitable for sensitive skin types, while also contributing to the physical stability of formulations containing labile active ingredients such as niacinamide, retinol, or hyaluronic acid. While used as a film-forming agent, HPMC forms a protective layer on the skin’s surface, promoting hydration and shielding against environmental aggressors [153]. This film also enhances the adherence and effectiveness of active compounds applied topically. Additionally, due to its gel-forming ability, HPMC enables the controlled or sustained release of active ingredients, such as salicylic acid, zinc PCA, or botanical extracts—thereby improving efficacy and reducing irritation potential. Owing to its inert and hypoallergenic nature, HPMC is also suitable for formulations intended for irritated, reddened, or atopic skin, contributing to soothing and protective effects without causing further sensitisation [154].

## 8. Conclusions

Hypromellose is a multifaceted ingredient that can serve as a film-forming agent in both thin films (with an active ingredient) and as a part of a coating film for APIs that have unpleasant organoleptic properties or to improve the swallowability of an ingredient. Several HPMC-derived compounds can be used to protect APIs from gastric juice due to the increased risk of instability in acidic media. As a film-forming agent in mucoadhesive films or ODFs, it usually improves the mechanical properties, with this parameter being directly connected to the amount of film-forming agents used. Since, in some cases, a fast disintegration and dissolution are recommended, a compromise needs to be made to achieve both a fast disintegration and good mechanical properties. In addition to these two main fields, HPMC can serve as a gel-forming agent, incorporating various active ingredients from different biopharmaceutical classes. Several APIs were incorporated into these matrices, serving as good tools for physicians in their daily activities. Another important role of HPMC is as a binder for the development of tablets with a modified release. In some cases, HPMC or other derived HPMC-compounds can be included in the tablet matrix (serving as a binder) or in the coating film, serving as a coating-forming film, but either way producing the API’s protection and intestinal release, which in many cases represents a challenge. Since it is a non-ionic cellulose-derived ingredient, the number of interactions with excipients is reduced only in extreme conditions (a very low or high pH or contact with oxidant ingredients), and its molecular weight and viscosity are affected.

Since HPMC is a versatile polymer and has multiple properties that can be used in drug delivery and pharmaceutical formulation, this excipient might serve as an ideal candidate for nanotechnology and nanocarriers, since it is biocompatible and biodegradable, and can stabilise nanoparticles. In addition to nanoparticles, HPMC can be used to develop 3D-printed tablets, with personalised dose, shape, and release profiles. Hypromellose can also be used for the oral delivery of peptides to improve their stability against oxidation and for extended-release formulations with proteins destined for subcutaneous or intramuscular injection. In conclusion, HPMC can serve as an environmentally friendly polymer that is preferred in vegan, animal-free, and nutraceutical products in addition to all the previously mentioned qualities.

In conclusion, the type of HPMC selected to develop thin films is of great importance since it can influence all the evaluated parameters in the case of these ODFs and mucoadhesive films. Frequently, if ODFs are developed, the HPMC E3, E5, and E15 are selected; while if mucoadhesive films are targeted, HPMC E50 can be a better solution. To accomplish all the critical quality attributes characteristic of thin films, combinations of film-forming agents might need to be taken into consideration alongside the amount of active ingredient, which in most cases does not exceed 100 mg, with this being the highest amount of active ingredient incorporated in a polymeric thin film up to this point.

## Figures and Tables

**Table 1:** Classification of HPMC types based on substitution grade.

Substitution Type		Methoxy (%)	Hydroxypropoxy (%)	Methocel^®^ Type *	Viscosity (mPa·s) *	Widespread Applications
1828		16.5 to 20.0	23.0 to 32.0	J	12,000, 75,000	Used as a thickener and rheology modifier.
2208		19.0 to 24.0	4.0 to 12.0	K	3, 100, 4000, 15,000, 100,000	Highly used for manufacturing controlled-release dosage forms, especially hydrophilic matrices, and also used for the development of capsule shells. HPMC 4M possesses mucoadhesive properties. HPMC K15M and K100M provide thickening and gelling properties.
2906		27.0 to 30.0	4.0 to 7.5	F	50, 4000	Binder, thickener, and rheology modifier. Commonly used in food, ceramics, coatings, and inks industries.
2910		28.0 to 30.0	7.0 to 12.0	E	3, 5, 6, 15, 50, 4000, 10,000, 15,000	HPMC E3, E5, E15, E50: popular representatives used as film-forming agents [32,33].
	Methoxy (%)	Hydroxypropoxy (%)	Methocel^®^ type *	Viscosity (mPa·s) *	Widespread applications.	
1828	16.5 to 20.0	23.0 to 32.0	J	12,000, 75,000	Used as a thickener and rheology modifier.	
2208	19.0 to 24.0	4.0 to 12.0	K	3, 100, 4000, 15,000, 100,000	Highly used for manufacturing controlled-release dosage forms, especially hydrophilic matrices, and also used for the development of capsule shells. HPMC 4M possesses mucoadhesive properties. HPMC K15M and K100M provide thickening and gelling properties	
2906	27.0 to 30.0	4.0 to 7.5	F	50, 4000	Binder, thickener, and rheology modifier. Commonly used in food, ceramics, coatings, and inks industries.	
2910	28.0 to 30.0	7.0 to 12.0	E	3, 5, 6, 15, 50, 4000, 10,000, 15,000	HPMC E3, E5, E15, E50: popular representatives used as film-forming agents [32].	

* The letters “J”, “K”, “F”, and “E” identify different HPMC products according to the Dow Chemical Company nomenclature.

**Table 2:** Commercial HPMC grades and their applications within the pharmaceutical industry.

Trademark Name	Substitution Type	Frequently Used Grades	Viscosity (mPa·s) *	Applications	Observations
Metolose^®^	2910	60SH	50–10,000	Thickening agent, manufacturing oral films.	The viscosity of the solution generated due to the water-soluble nature of the polymer depends on the amount and grade used.
	2906	65SH	50–4000	Used as a binder, thickener and film-forming agent in solid dosage forms.	
	2208	90SH	4000–100,000		
	2208	90SH-SR	100SR 4000SR 15000SR 100000SR	Sustained-release dosage forms for oral use—suitable for direct compression and wet granulation.	It is designed exclusively for a hydrophilic matrix agent.
Pharmacoat^®^	2910	603	3	Pellet coating, for binder solutions used in wet granulation.	Low viscosity HPMC substitution grades. Developed in 1963, first used for film coating and later for manufacturing capsules in the place of gelatin or as a binder for granulation [40].
		645	4.5	Manufacturing capsule shells and amorphous solid dispersions.	
		606	6	Tablet coating, manufacturing capsule shells, oral films.	
		615	15	Taste masking by coating with enteric polymer and pore former, coating fragile tablets.	

* measured in 2 wt.% aqueous solution at 20 °C according to the United States Pharmacopeia measuring method.

**Table 3:** Active ingredients incorporated in orodispersible films, where HPMC served as a film-forming agent (randomized selection).

HPMC Type	Concentration	Active Ingredient	Method (s)	Observations	References
HPMC E5, HPMC E15	10–20%	Phenytoin (30 mg)	Syringe extrusion—3D printing	20% (*w*/*v*) HPMC E5 and 10% (*w*/*v*) HPMC E15 suitable for the drug incorporation and 3D-printing. By loading the phenytoin into the HPMC E15 matrix, good physical appearance, good mechanical strength, rapid disintegration time, and a fast release of the API were obtained.	[109]
HPMC E15	10%	Mirtazapine	Solvent casting method 3D-printing method	Through both methods, an amorphous form was obtained in the case of the final mixture. The 3D-printing method outlined a porous matrix, good mechanical properties, a fast disintegration time, and an immediate release of mirtazapine (>80% in five minutes).	[98]
HPMC (Colorcon)	3%	Meloxicam (5.16–5.51 mg)	Solvent casting method	ODFs with 3% HPMC and 2% microcrystalline cellulose and 3% HPMC and 2% Kolidon^®^ were prepared. Disintegration times higher than 30 s for all the formulations developed; amounts of API < 60% were released at 120 min in comparison to the formulations where sodium alginate was used, and almost 80% of the active ingredient was released at the same time frame.	[102]
HPMC E5	8–9%	Caffeine	Solvent casting method	Four formulations coded CAF1 (8% HPMC E5, only caffeine), CAF2 (8% HPMC E5, caffeine + citric acid [1:1]), CAF3 (9% HPMC E5, caffeine + citric acid [1:1]), and CAF 4 (9% HPMC E5, caffeine + sodium benzoate [1:1]. Very fast release of the active ingredient, especially in the CAF4 formulation, where sodium benzoate was used as a hydrotropic ingredient (almost 100% of API release at 3 min). Disintegration times lower than 180 s were noticed in the case of both methods used (slide-frame method and basket-rack method). CAF2 and CAF3 exhibited good mechanical properties, with CAF2 outlining the highest number of folds needed to produce the rupture of the film, and similar tensile strengths for CAF2 and CAF3.	[103]
HPMC E5	20%	Loratadine	3D printing	The amorphous state was outlined through a DSC study, which highlighted the lack of the endothermic peak that was obtained in the case of loratadine in its crystal form. Loratadine-ODFs were obtained with an average mass of 60.22 ± 5.02 mg, 164 ± 15 µm, and a disintegration time lower than the maximum admitted by the Ph. Eur. 11 for ODTs (180 s) of 146 ± 23 s.	[101]
HPMC—viscosity 28 cP (2% in H_2_ O)	2%	Clove oil (nano-sized)	Solvent casting method	The influence of additives maltodextrin, pectin, and glycerol on the ODF was studied. A decrease in tensile strength and an increase in elongation at break and opacity were observed in clove oil ODFs compared to blank HPMC-ODFs.	[110]
HPMC E15 and maltodextrin	3–5%	Mosapride citrate	Solvent casting method	Mosapride citrate solid dispersion with poloxamer 188 was incorporated in ODFs. To establish an optimal formulation, the type of plasticiser, its concentration, and the ratio between the selected film-forming agents were varied. Thicknesses between 0.17 ± 0.09 (F20: 15% propylene glycol and 1:9 maltodextrin–HPMC ratio) and 0.35 ± 0.08 mm (25% propylene glycol and 5:5 maltodextrin–HPMC ratio). Good mechanical properties in terms of tensile strength ranging between 2.6 MPa for F12 (25% glycerol and 5:5 maltodextrin–HPMC ratio) and 29.9 MPa F8 (15% glycerol and 5:5 maltodextrin–HPMC ratio). Elongation varied between 17.3% F13 (15% glycerol and 3:7 maltodextrin–HPMC ratio) and 82.8% in the case of F12, whose composition is mentioned above. Fast dissolution rates were registered in most of the formulations, with the formulation that contained 25% glycerol and a 3:7 maltodextrin–HPMC ratio exhibiting the fastest amount of API release.	[111]
HPMC viscosity of 6 mPa and HPC mixtures	20–90%	Donepezil hydrochloride	Solvent casting method	Different blends of HPMC and HPC were used to develop ODFs. The 40/60 and 20/80 HPMC/HPC ratios outlined lower tensile strength and elongation, whilst blends > 40% HPC exhibited shorter disintegration times. The addition of donepezil hydrochloride reduced tensile strength and elongation.	[112]
HPMC (Pharmacoat^®^ 606) and three types of HPC	12.5–17.5/ 10/15	Loperamide hydrochloride (LPH) and Ibuprofen	Solvent casting method	Dispersions were characterised in terms of viscosity and particle sedimentation, whilst the ODFs were verified for uniformity of content, thickness, mass, and stability. ODF obtained from low viscous suspensions, loaded with 10 mg/6 cm^2^ LPH/film, while the ones obtained from high viscous suspensions were loaded with up to 5 mg LPH/6 cm^2^ film. ODFs with 50 mg/6 cm^2^ IBU resulted in acceptable parameters, while higher concentrations of IBU in ODFs were less feasible.	[113]
HPMC E5, PVP K30 and maltodextrin	8–12%	Fluoxetine	Solvent casting method	Twelve formulations of ODFs (six blank and six with fluoxetine) were developed by varying the amount of HPMC (between 8 and 12% *w*/*w*) with good mechanical properties and good disintegration ability. FX1 and FX2 (10% HPMC E5 with different amounts of PG 10% for FX1 and 12% FX2) released >97% of fluoxetine within 15 min.	[114]
HPMC E50, HPC	5%	Herpetrione (nanosuspension)	Solvent casting method	Disintegration time < 30 s with reconstituted nanosuspension particle size of 280 ± 11 nm. Good redispersibility of the herpetrione ODFs. ODF containing herpetrione nanoparticles, an opportunity to transform drug nanosuspensions into a solid dosage form. ODFs with nano-sized API enhancing the dissolution rate of poorly water-soluble drugs.	[115]

**Table 4:** Examples of APIs used in different HPMC pharmaceutical formulations.

Nr.	Active Pharmaceutical Ingredient	Formulation Type	Application	Reference
1	Azelaic acid	hydrogel	acne	[116]
2	Azithromycin	nanoparticle	ocular infections	[117]
3	Bupivacaine	in situ gel	ocular anaesthetic	[118]
4	Buspirone	buccal discs	anxiety	[119]
5	Chloramphenicol	gel	ocular	[120]
6	Clozapine	bilosomal gels	schizophrenia	[121]
7	Duloxetine	cyclodextrin	depression	[122]
8	Enalapril	floating tablet	hypertension	[123]
9	Fluconazole/ofloxacin	mucoadhesive film	polymicrobial keratitis	[124]
10	Gliclazide	nanosuspension	diabetes	[125]
11	Insulin	nanogel	diabetes	[126]
12	Losartan	transdermal patch	hypertension	[127]
13	Metformin	floating tablet	diabetes	[128]
14	Progesterone	mucoadhesive tablets	reproductive therapy	[129]
15	Quetiapine	tablets	Bipolar disorder	[130]
16	Rosuvastatin	tablet	hypercholesterolemia	[131]
17	Tenofovir	bioadhesive gel	HIV	[132]
18	Timolol/Brimonidine	liposome	glaucoma	[133]
19	Tranexamic acid	gel	local haemorrhages	[134]
